# The feasibility of community level interventions for pre-eclampsia in South Asia and Sub-Saharan Africa: a mixed-methods design

**DOI:** 10.1186/s12978-016-0133-0

**Published:** 2016-06-08

**Authors:** Asif Raza Khowaja, Rahat Najam Qureshi, Diane Sawchuck, Olufemi T. Oladapo, Olalekan O. Adetoro, Elizabeth A. Orenuga, Mrutyunjaya Bellad, Ashalata Mallapur, Umesh Charantimath, Esperança Sevene, Khátia Munguambe, Helena Edith Boene, Marianne Vidler, Zulfiqar A. Bhutta, Peter von Dadelszen, CLIP Working Group

**Affiliations:** Division of Women & Child Health, Aga Khan University, Karachi, Pakistan; Department of Obstetrics and Gynaecology, and the Child and Family Research Institute, University of British Columbia, Vancouver, Canada; Centre for Research in Reproductive Health (CRRH), Olabisi Onabanjo University Teaching Hospital, Sagamu, Ogun State Nigeria; KLE University’s JN Medical College, Belgaum & SN Medical College, Bagalkot, India; Manhiça Health Research Centre (CISM), Mozambique and Faculty of Medicine, Universidade Eduardo Mondlane (UEM), Maputo, Mozambique

**Keywords:** Community-based interventions, Pre-eclampsia, Eclampsia, Methodology, Feasibility study

## Abstract

**Background:**

Globally, pre-eclampsia and eclampsia are major contributors to maternal and perinatal mortality; of which the vast majority of deaths occur in less developed countries. In addition, a disproportionate number of morbidities and mortalities occur due to delayed access to health services. The Community Level Interventions for Pre-eclampsia (CLIP) Trial aims to task-shift to community health workers the identification and emergency management of pre-eclampsia and eclampsia to improve access and timely care. Literature revealed paucity of published feasibility assessments prior to initiating large-scale community-based interventions. Arguably, well-conducted feasibility studies can provide valuable information about the potential success of clinical trials prior to implementation. Failure to fully understand the study context risks the effective implementation of the intervention and limits the likelihood of post-trial scale-up. Therefore, it was imperative to conduct community-level feasibility assessments for a trial of this magnitude.

**Methods:**

A mixed methods design guided by normalization process theory was used for this study in Nigeria, Mozambique, Pakistan, and India to explore enabling and impeding factors for the CLIP Trial implementation. Qualitative data were collected through participant observation, document review, focus group discussion and in-depth interviews with diverse groups of community members, key informants at community level, healthcare providers, and policy makers. Quantitative data were collected through health facility assessments, self-administered community health worker surveys, and household demographic and health surveillance.

**Results:**

Refer to CLIP Trial feasibility publications in the current and/or forthcoming supplement.

**Conclusions:**

Feasibility assessments for community level interventions, particularly those involving task-shifting across diverse regions, require an appropriate theoretical framework and careful selection of research methods. The use of qualitative and quantitative methods increased the data richness to better understand the community contexts.

**Trial registration:**

NCT01911494

**Electronic supplementary material:**

The online version of this article (doi:10.1186/s12978-016-0133-0) contains supplementary material, which is available to authorized users.

## Background

Globally, hypertensive disorders of pregnancy (HDP) mainly pre-eclampsia and eclampsia are major contributors to maternal and perinatal mortality and morbidity, with the highest burden is in low and middle-income countries (LMIC) [1, 2]. Management of pre-eclampsia and eclampsia has focused on hospital-based interventions [3], and the only intervention possible at the community level is stabilization and referral to higher-level facility [4]. As these conditions are dependent on timely and appropriate intervention, many women in hard-to-reach areas suffer from severe disability or death as a result of delays in early identification, triage, transport and treatment [5]. To effectively reduce maternal and perinatal complications resulting from pre-eclampsia and eclampsia, community-level identification, prompt intervention and referral are required [6]. Therefore, a package of evidence-based interventions that are applicable in the home and primary health centre (PHC) represents a critical step towards addressing excess maternal and perinatal deaths and disabilities resulting from the failure to identify and rapidly manage pre-eclampsia and eclampsia at the community level. Such a package would require community health care providers to use a simplified triaging tool to identify women at high risk of adverse outcomes, provide emergency treatment and facilitate their referral to hospital. A systematic review of strategies to improve maternal and perinatal health in LMICs demonstrated the benefits of using such community-based interventions for improving maternal and newborn outcomes [7].

### The Community Level Interventions for Pre-eclampsia (CLIP) trial

The CLIP Trial [clinicaltrials.gov number ID NCT01911494] is an ongoing cluster randomized controlled trial, which aims to address the maternal and perinatal mortality resulting from the failure to identify and rapidly manage pre-eclampsia and eclampsia at the community level in LMICs [8]. Specifically, the CLIP intervention consists of:

I. *Community engagement* including women from the communities, dyadic household decision-makers (husbands, fathers-in-law) and community leaders about: pre-eclampsia, its origins, symptoms, signs and potential consequences, pre-permissions for maternal transport and fundraising activities for transport and treatment costs;
II. *Provision of HDP-oriented antenatal care through household visits* by community healthcare providers (cHCPs) who carry a mobile health (m-health) application for identifying women at risk of pre-eclampsia. The m-health application is programmed with a validated Pre-eclampsia Integrated Estimate of Risk (PIERS) on the Move (POM) [9, 10];
III. *Use of the CLIP package for women with a CLIP ‘trigger’* (i.e. oral antihypertensive therapy or intramuscular (i.m.) magnesium sulphate (MgSO_4_) when indicated, and appropriate referral to a comprehensive emergency obstetric care (CEmOC) facility as needed).
IV. Capacity building through continuous medical education of healthcare providers in referral health facilities.

The cHCPs assess pregnant women with a target frequency of every 4 weeks at a minimum. These visits can occur in the home or PHC. The cHCPs are trained to enquire about the woman’s symptoms (using country-specific pictograms), take blood pressure and check urine for protein to inform diagnosis of and risk assessment for pre-eclampsia. The control group (without intervention) continues with routine pregnancy care.

### Feasibility assessment of the CLIP trial

**Fig. 1:**
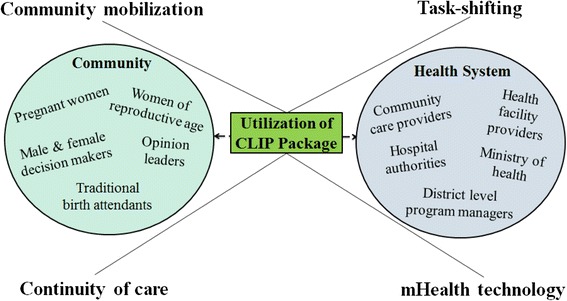
Stakeholders of the CLIP feasibility study. CLIP, Community Level Interventions for Pre-eclampsia

Implementation of the complex CLIP interventions significantly depended on positive interactions with the community and existing health system (Fig. 1). The CLIP Trial is recruiting in four countries, which have country-specific healthcare delivery systems, diverse population characteristics, varied perceptions of care seeking and treatment preferences. Therefore, a prior assessment of the acceptability and feasibility of the CLIP interventions was needed to ensure effectiveness, while addressing applicability and sustainability issues relating to implementing the trial.

Very few clinical trials [11, 12], particularly in the area of pre-eclampsia and eclampsia [13, 14], have conducted feasibility assessments prior to initiating large-scale community-based interventions. Failure to fully understand the study context risks the effective implementation of the intervention and limits the likelihood of post-trial scale-up. Consequently, in the absence of a feasibility assessment the benefits of the intervention may not be maximized at population level, despite being shown to be effective. Therefore, the role of such a feasibility study was integral to successful trial implementation and programmatic sustainability.

### Feasibility study objectives

The feasibility assessment of the CLIP Trial aimed to describe the health system, identify community and individual barriers and facilitators that influence care of pregnant women in the community, particularly as they relate to pre-eclampsia and eclampsia, in preparation for the conduct of a community-based cluster randomised trial. The primary objectives of the CLIP Trial feasibility study were to:

1. Explore the local cultural beliefs related to pregnancy and its complications
2. Understand current care seeking behavior and practices
3. Identify local stakeholders and determine support
4. Establish rates of maternal and perinatal mortality and morbidity in study communities to confirm sample size

Secondary objectives were to assess each health care system including organization, infrastructure and human resource capacity, cost of maternity services, and training needs for health care providers.

## Methods

### Theoretical framework

The study design was guided by May et al.’s normalization process theory (NPT), as it has been used in designing, implementing and evaluating trials of complex clinical interventions [15, 16]. As the CLIP Trial aims to integrate an innovative and complex healthcare intervention into regular practice, the NPT theoretical framework was an appropriate fit [17].

NPT emphasizes on the collective work of individuals and groups to make the intervention normalized. Particularly, it seeks to understand the context that increases or decreases the likelihood of adoption of an intervention into existing system. There are four key components to NPT including coherence, cognitive participation, collection action and reflexive monitoring [18]. The application of NPT key components guided CLIP feasibility study as follows:

(I) *Coherence*: This component refers to aspects of a complex intervention that are similar to existing practice. The CLIP introduces m-health technology that builds on the existing infrastructure of cHCP and enhances health system capacity through additional trainings. It also implies cooperative interaction between cHCP and collective effort to integrate m-health into current practices. This assessment required review of health workers’ curriculum, practice guidelines and policies.
(II) *Cognitive participation*: This component refers to understanding the dynamics of intervention and potential benefits/risks from participation. The CLIP Trial proposes immediate and long-term benefits to the community and health system. The qualitative methods in the CLIP feasibility study provided an opportunity to discuss in detail importance and potential benefit/risk with wide range of stakeholders.
(III) *Collective action*: This component refers to collaboration between individuals and groups responsible to implement intervention. The implementation of the CLIP Trial highly depended on the collective action of all stakeholders (i.e., care providers, care receivers and community at large). The collection action was gauged through discussion of participatory activities, such as community engagement, capacity building of healthcare providers, and on-going support of research staff.
(IV) *Reflexive monitoring*: This component refers to reflecting upon the enabling and impeding factors that could potentially normalize the intervention. We used reflexive monitoring for researchers to provide feedback during data collection and to assess the level of community and stakeholder support. Reflexive monitoring was also done throughout FGDs and interviews through the use of paraphrasing by facilitators for community and individual appraisal of benefit.

### Study design

**Fig. 2:**
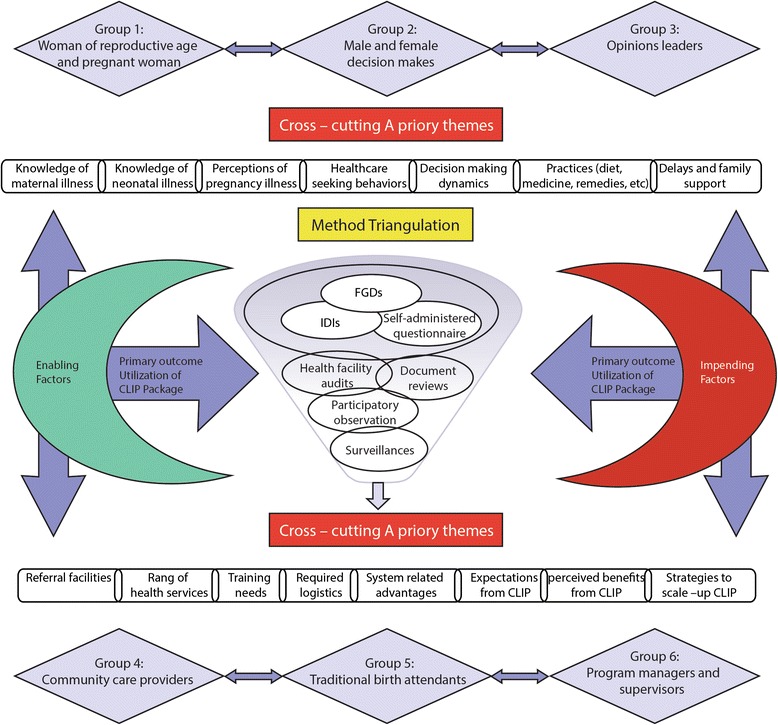
Snapshot of mixed-method design for the CLIP feasibility study. CLIP, Community Level Interventions for Pre-eclampsia; FGDs, focus group discussions; IDIs, in-depth interviews

This study used a pragmatic mixed-methods (qualitative-and-quantitative) design informed by the theoretical framework NPT. The quantitative and qualitative components were used to complement one another and allow for triangulation between methods (Fig. 2) [19, 20].

### Study sites

**Fig. 3:**
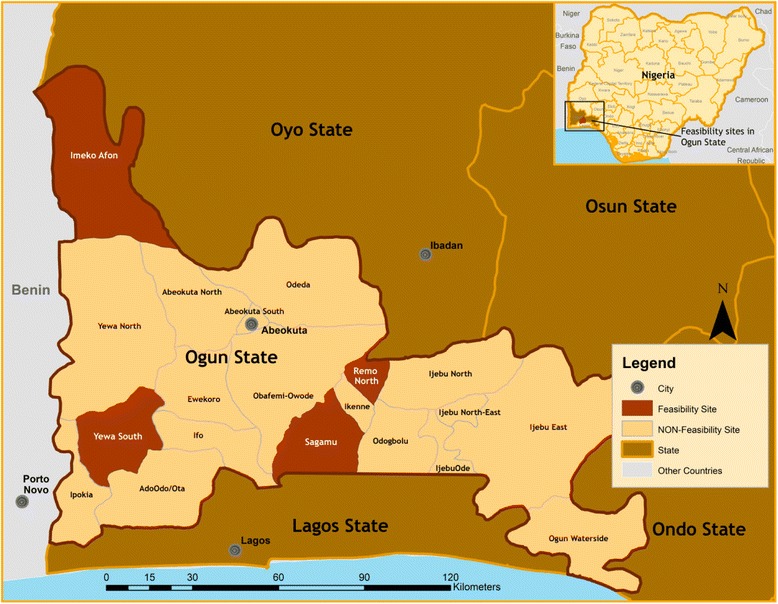
Study site map of Nigeria

**Fig. 4:**
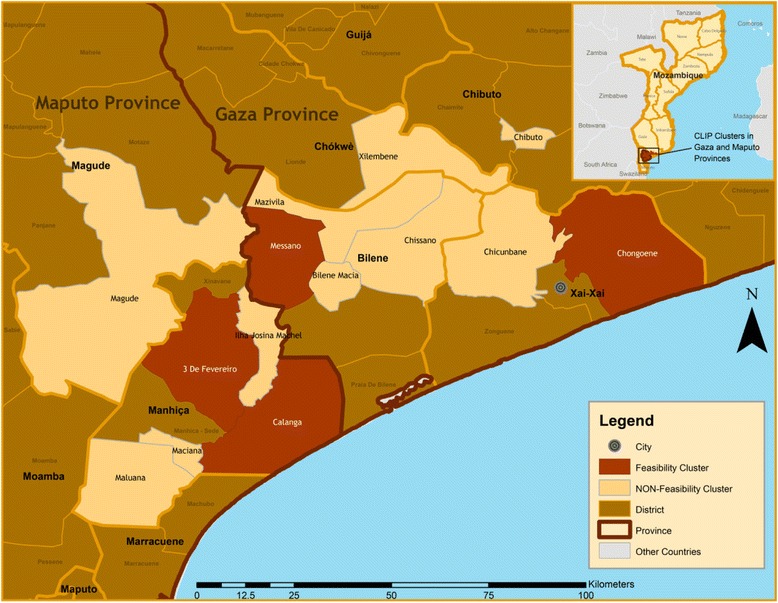
Study site map of Mozambique

**Fig. 5:**
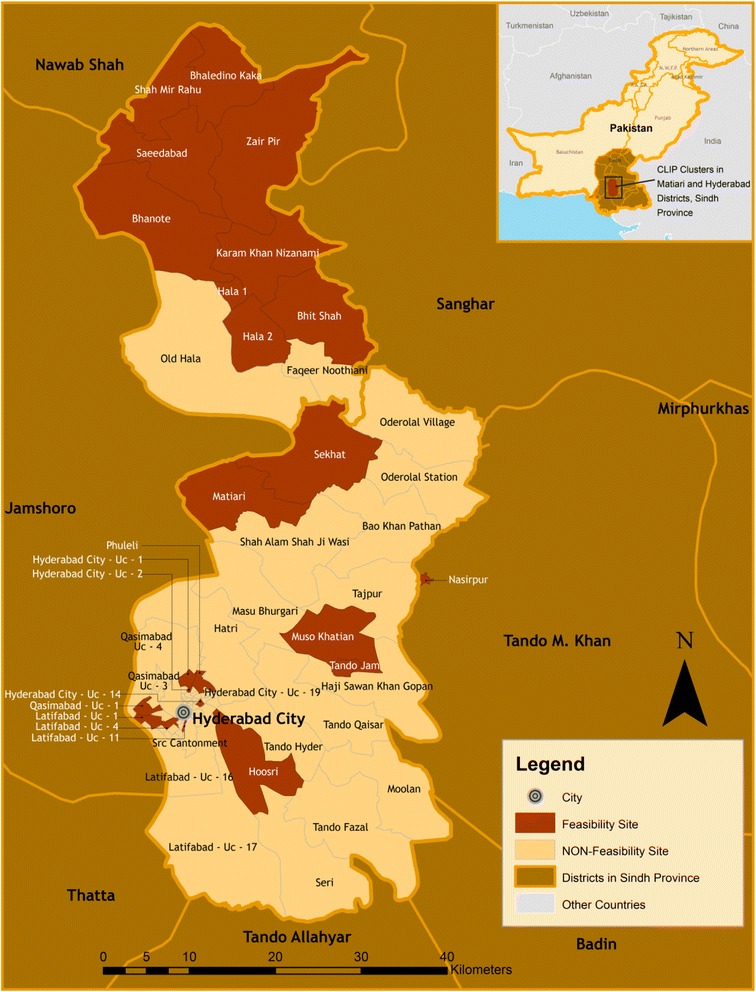
Study site map of Pakistan

**Fig. 6:**
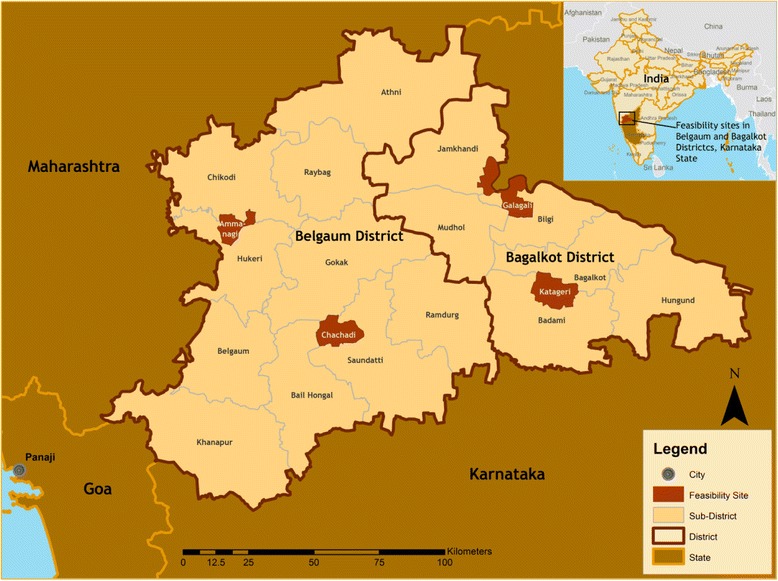
Study site map of India

Four countries were selected for this feasibility study: Nigeria (Fig. 3), Mozambique (Fig. 4), Pakistan (Fig. 5), and India (Fig. 6). In-country activities were led by: the Centre for Research in Reproductive Health (CRRH) in Nigeria; the Manhiça Health Research Centre (CISM) in Mozambique; the Aga Khan University (AKU) in Pakistan; and KLE University’s JN Medical College and the SN Medical College in India. These sites were selected in consultation with lead organizations in each country, as well as existing academic relationships, understanding and experience of community-based maternal or perinatal health research work, and research infrastructure.

### Study duration

The feasibility studies were completed in Pakistan during February 2012 to May 2013; India during September 2012 to August 2013; Mozambique during July 2013 to February 2014; and Nigeria during February 2012 to May 2013.

### Data collection: qualitative methods

Ethnography is a methodology born out of the field of anthropology [21]. This qualitative approach is used to explore differences and similarities between cultural groups. The underlying assumption is that several differing perceptions or understandings exist and that these are socially constructed, they are influenced by their cultural group and subject to future change [22]. Therefore, an ethnographic lens is often adopted when researchers aim to explore beliefs and practices related to a particular phenomenon within or between groups.

As our study aimed to describe the knowledge, attitudes and behaviours of various cultural groups, ethnography was an appropriate methodological approach. In our study, we were interested in how various cultural groups and communities make sense of pregnancy and pregnancy complications with a particular focus on pre-eclampsia. In addition, we explored how the cultural group of ‘health care providers’ act in the face of pregnancy complications, particularly pre-eclampsia.

The following methods were used to understand culturally based practices and their underlying factors.

#### Focus Group Discussions (FGDs)

FGDs were conducted to elucidate in-depth information and to encourage group dialogue. This method allowed researchers to select and engage a large number of participants from varied groups. FGDs were preferred to explore the collective experiences of community members, health care providers, and policy makers in parallel groups.

One researcher, assisted by a second to record field notes and audio recordings, facilitated all FGDs. Moderators were local researchers with backgrounds in community or obstetric medicine or qualitative researcher. Facilitators were locally recruited based on their research experience, community knowledge, familiarity with the health care system, and qualitative research expertise. All researchers were provided basic qualitative training prior to data collection; the process was supervised by at least one social scientist at each site. To respect local preferences of participants, FGDs were held separately for men and women in all countries. In addition, facilitators were most often of the same gender as participants. FGD facilitators had high proficiency in all locally spoken dialects.

There was no known relationship between respondents and participants; facilitators did not provide health care services for any participants. Rapport building with communities was essential in each site prior to data collection. Approval was obtained by community leaders and chiefs prior to conduct of the study. All participants were briefed on the study prior to providing written informed consent.

Participants were considered eligible for the study if they expressed availability for at least 60 min and a willingness to participate. A non-probability sampling approach was taken; study participants identified through the primary health centre networks, as well as local community workers. Women of reproductive age were defined as women 15–49 years (except in Mozambique where reproductive age was considered to be 12–49 years). In India, male and female decision-makers were approached for participation when they accompanied women of reproductive age to the health centre. Culturally appropriate yet feasible strategies were employed to approach, invite, and ensure participation of stakeholders at respective sites. These were designed to strengthen the rapport with the community in a way that enabled participants to benefit from participation. One benefit was for participants to freely discuss issues related to pregnancy in the community. Participants were approached by phone or face-to-face. Each FGD included an average of 7 to 10 participants. No incentives were given for participation in FGDs; however refreshments and transport costs were provided in some sites. Project staff arranged refreshments and provided children with group play during the FGDs. The desired number of FGDs was determined by data saturation, and data collection stopped when saturation was reached at each site [23]. The FGDs were transcribed verbatim in local language and translated for analysis. A total of 123 FGDs were completed, as determined by data saturation (Table 1).

**Table 1:** Distribution of focus group discussions at respective study sites

Level	Focus groups	Sites				Total
		Mozambique	Nigeria	Pakistan	India	
Community	Women of reproductive age, and pregnant women	5	16	19	5	45
	Male and Female decision makers (husband/partners, father in-law, & mother in-law)	10	4	7	6	27
	Opinion/religious leaders or community stakeholders	1	4	-	2	7
	Health committees	1	-	-	-	1
Care provider	Community health care providers	5	7	7	4	23
	Medical officers, and obstetricians	-	1	-	1	2
	Nurses and midwives	-	4	-	4	8
	Faith-based care providers	-	1	-	-	1
	Traditional birth attendants	5	4	-	-	9
Total		27	41	33	22	123

Centrally located venues were chosen to best accommodate participants: primary health centres, local households, and other community gathering locations. Non-participants were not present during FGDs with the exception of small children in some cases. Community perspectives were obtained from women of reproductive age (represented by pregnant women and mothers of children under five years of age), opinion leaders, religious leaders, village community leaders, husbands, and male and female decision-makers (including family members, and particularly mothers-in-law). In addition, FGDs were conducted with community health workers who were represented by various cadres in the four countries: Community Health Extension Workers (CHEW) in Nigeria, Agente Polivalente Elementar (APE) in Mozambique, Lady Health Workers (LHW) in Pakistan, and Accredited Social Health Activists (ASHA), staff nurses and Auxiliary Nurse Midwives (ANM) in India. In addition, a variety of other health care providers were included given their key role in health care service delivery: medical officers, obstetricians, faith-based providers, and traditional birth attendants. These groups were chosen to represent the breadth of the communities’ views.

FGD guides were developed from the literature and broadly categorized as knowledge and perceptions of pregnancy [24, 25], maternal care seeking practices [26], household and community dynamics [27, 28], use of alternative medicines and providers [29], as well as the cost and availability of health care services and transport [30]. The guides were translated into the local language in all sites to best promote interaction with the community members and obtain the richest data: Yoruba in Nigeria, Changana and Portuguese in Mozambique, Sindhi and Urdu in Pakistan, and Kannada and Marathi in India. Guides underwent pilot testing for content validity review by each country-specific research team, and questions were adapted for cultural sensitivity and local use. FGD guides developed for the study were semi-structured to promote a natural discussion progression.

#### In-depth interviews

Interviews allowed rich in-depth data collection from individuals [31]. Interviews were utilized for stakeholders for whom convening groups was not always either feasible or appropriate. This included health care providers, opinion leaders, and policy makers.

Interviewers were responsible for facilitating the discussion, recording field notes and audio. These researchers were familiar with the study and received training on qualitative data collection, qualitative data management, ethical conduct and discussion guides. Many facilitators had a medical background with experience in community medicine or obstetrics, while the rest were qualitative researchers. Facilitators were locally recruited based on their research experience, community knowledge, familiarity with the health care system, and qualitative research expertise. They were both men and women, and none were the direct health care providers or supervisors of participants. Approval for interviews was obtained by the relevant community leaders or supervisors as needed. All participants were briefed on the study prior to providing written informed consent.

The participants were considered eligible for interviews if they expressed availability for at least 45 to 60 min, a willingness to participate, and met the desired stakeholder description. A purposeful sample was used in all sites, where eligible participants were selected with the help of community representatives and hospital administration, and were contacted in person or by phone. All interviews were conducted privately, one-to-one, which ensured no undue pressure or discomfort for participants. Data collection was done at a place suitable for the interviewees. Participants were provided compensation for their time in some sites, while others provided transport and refreshments only. The desired number of interviews was determined by data saturation, and data collection stopped when saturation was reached at each site [23]. The interviews were transcribed verbatim in local language and translated for analysis.

A total of 100 interviews were completed, as determined by the data saturation (Table 2).

**Table 2:** Distribution of in-depth interviews at respective study sites

Level	In-depth informants	Sites				Total
		Mozambique	Nigeria	Pakistan	India	
Policymakers	Opinion leaders, and community stakeholders	-	4	-	-	4
	Head of local government and programme directors	-	7	-	-	7
	Hospital administration and supervisors of community health workers.	3	12	10	-	25
Care providers	Medical doctors, specialist/SOG member, obstetricians, reproductive-child health officers, and private practitioners	5	11	9	12	37
	Traditional birth attendants or traditional healers	5	5	7	-	17
Community	Local NGO representatives	5	-	-	-	5
	Knowledgeable women/matrons	5	-	-	-	5
Total		23	39	26	12	100

Interviewees included medical officers and obstetricians from the public and private health care system. Traditional health care providers, including female elders and traditional birth attendants, were interviewed in all sites, except India. Various community and facility-level policy makers were interviewed: local government representatives, non-governmental organization representatives, hospital administrators, and community leaders.

Interview guides were developed for this study based on key constructs and themes of interest: health care related experience, obstetric knowledge, treatment practices, access to health care services, and health care provider and community dynamics.

Interviews were conducted in a variety of languages – English and Yoruba in Nigeria, Portuguese and Changana in Mozambique, Sindhi and Urdu in Pakistan, and English in India. Guides were pilot tested and modified based on input from the research team and the field-test prior to use. Semi-structured interviews allowed facilitators to tailor questions and probes to the context and participants. The sessions began with broad questions to initiate discussion. Some sensitive questions related to maternal deaths were included; to mediate the possible negative feelings associated with this discussion they were placed at the end.

#### Participant observations

In Nigeria, observations were conducted for one full antenatal clinic day in four primary health centres. A community-based researcher was responsible for observing and recording the field notes during these observations.

#### Document review

Community health care providers deliver basic maternal and child health services at the door step in many LMICs [32, 33]; however, their training and experience varies widely between countries [34, 35]. Systematic reviews of community health worker training curricula, job descriptions, and practice guidelines were conducted in all four countries to determine the community health workers’ scope of practice and ability to participate in the CLIP Trial. In addition, a review was conducted of regional and facility-based policies and guidelines for the management of pre-eclampsia. Finally, any regional or national policies related to community health worker provision of maternal services in country was reviewed and summarized.

### Data collection: quantitative methods

Quantitative methods were employed to determine community health care provider training needs, competence and skills, as well as health care system organization and infrastructural capacity in study areas.

#### Health facility assessment

Data collection tools for health facility capacity and resource availability were based on the published literature and existing guides related to healthcare system resources and capabilities for maternal, obstetric and neonatal care in the context of developing countries [36, 37]. The information collected matches in many areas to the World Health Organization’s identified ‘service readiness indicators’ [38]. In addition, facility assessment tools used in Mozambique were informed by the 2012 Service Provision Assessment (SPA) survey [39]. The scope of this assessment was to identify the availability of basic, as well as comprehensive emergency obstetric care (CEmOC) facilities, diagnostic services, staffing, working hours, health facility utilization (outpatient/in-patient visits), referral points, cost of care, and maternal mortality. Surveys underwent modifications in each country before translation. All data collection tools underwent pilot testing for content validity review by each country-specific research team and questions were adapted accordingly. Primary, secondary and tertiary level health care facilities were surveyed after obtaining consent on site. In addition, pharmacies were surveyed (where relevant) to identify the availability and cost of essential maternal and newborn commodities (Table 3).

**Table 3:** Health facilities surveyed

Facilities surveyed	Sites				Total
	Mozambique	Nigeria	Pakistan	India	
Public primary/secondary health facilities	54	47	14	17	132
Public tertiary care health facilities	2	1	1	2	6
Private secondary/tertiary health facilities	-	16	12	65	93
Laboratories	-	-	25	-	25
Drug stores/Pharmacies	-	-	81	-	81
Total	56	64	133	84	337

#### Community health care provider questionnaire

Data were collected through self-administered questionnaires in Pakistan (LHWs), India (ANMs), and Mozambique (APEs). Nigeria did not utilize this data collection method (Table 4).

**Table 4:** Self-administered health care provider questionnaires

Community health care providers	Country	Numbers
Lady health workers	Pakistan	458
Auxiliary nurse midwives	India	8
Staff nurses	India	2
Agente Polivalente Elementar	Mozambique	81
Total		549

All community health care providers who were reachable at the time of data collection in the study areas were approached for participation. Some health care providers were on leave or otherwise unreachable at the time of data collection and therefore were not included. Participants were briefed on the questionnaire purpose and general content prior to informed consent. Research staff coordinated with district health authorities and supervisors to recruit community health care providers. Questionnaires were completed and collected on the same day or within a week time, as feasible. Questionnaires were designed to obtain information concerning health worker knowledge and skills to manage pregnant women and to perform home-based treatment for women with pre-eclampsia. Questions used a five-point Likert scale. This format was appropriate for the large sample size and to reflect participants’ attitudes. None of the Likert questions were negatively worded. In addition, one open-ended question was included at the end to allow written responses with greater elaboration if desired. Data collection was done in the health facility or home to minimize the required time commitment and maximize convenience. The data collector did not intervene during the questionnaire unless clarification was requested.

#### Baseline household demographic and health survey

Individual and household level surveys were undertaken in all study areas. The primary objective of this survey was to establish baseline rates of maternal and perinatal mortality and morbidity in study communities to confirm sample size. Second, this survey enabled researchers to beta-test surveillance tools and data management prior to the trial. The key variables on the baseline survey questionnaire included household socio-demographic information, obstetric and general medical history over the previous 12 months. The women of reproductive age, living in the study catchments, and willing to participate in the study were considered eligible for the survey. In Nigeria, Mozambique and Pakistan individual data collection was performed by trained medical and/or research staff. In India, these data were collected prospectively over a 5-month period (Table 5).

**Table 5:** Estimated sample size for baseline survey at the community

Country	Numbers of households/women
Pakistan	88,410 households
Nigeria	32,042 households
India	5189 women
Mozambique	50,493 households
Total	

### Data quality control

Stringent quality control measures were employed at each site. This included field supervisors and senior social scientists who undertook spot visits to observe data collection procedures. Photographs, audio recordings, field site data checks, peer debriefing, real-time data entry, and computer-assisted data analysis were also used to maintain data quality. Reflexivity and data triangulation are widely cited methods of ensuring rigor and quality control in the qualitative research [40, 41]. In this study, the data collectors undertook both self and group reflections after FGDs and interviews. These reflections and debriefing were instrumental in contextualizing the data, as well as ensuring a transparent process. Data triangulation between multiple methods of data collection was helpful to validate information from a diverse range of participants.

Project management and oversight were the responsibility of the central CLIP Co-ordinating Centre at the University of British Columbia, in collaboration with the local principal investigators. Collaboration took the form of frequent email communication, teleconferences, and site visits.

### Data management and analysis

#### Qualitative data

**Fig. 7:**
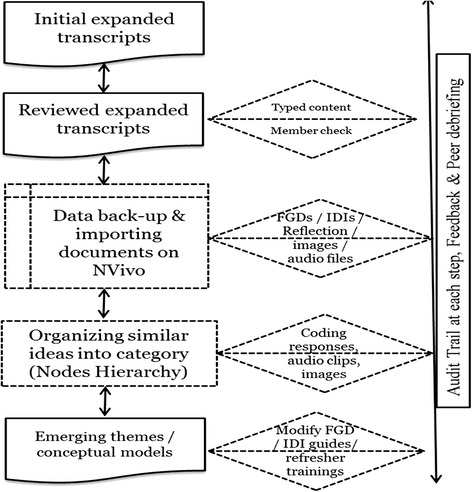
Steps of qualitative data analysis using QSR NVivo-10. FGD, focus group discussion; IDI, in-depth interview

Digital voice recorders and hand written field-notes were used to record discussions during focus group discussions and interviews. Analysis was conducted in Sindhi in Pakistan, in English in India and Nigeria, and in a combination of English and Portuguese in Mozambique. All translations were confirmed by multiple researchers with back-translation of data segments for quality control. Each FGD and interview was assigned a unique identification number, and photographs taken during data collection and reflection notes were attached to transcripts for analysis. The number of data coders varied by country: one in Pakistan, one in India, one in Nigeria, and two in Mozambique. All coded transcripts in India and Nigeria were cross-checked by the local research team to resolve or clarify any misinterpretation of the data. Thematic analysis (combining inductive and deductive approaches) was performed in country by the local country team or analysis was supported by the central trial team, as required. Using deductive reasoning, the results were grouped into predetermined categories of key themes related to the discussion guides. During analysis, inductive reasoning was used to incorporate new and unexpected ideas. This produced a comprehensive analysis structure to reflect the richness and variety of responses. Data were analysed using NVivo 10 software (Fig. 7).

#### Quantitative data

Data consistency checks were established in the data entry software. Data were double entered in real time, and cleaned prior to analysis. SPSS 19 (IBM, Armonk, NY, USA) Epi Info 7 [CDC, Atlanta, GA, USA], or Stata 13 (Stata Corp. College Station, Texas, USA) were used to calculate frequencies and proportions.

### Ethics approval

The CLIP Trial was registered at Clinicaltrials.gov. The Clinical Research Ethics Board of the University of British Columbia, Vancouver Canada, approved the CLIP Trial feasibility work (ETHICS # H12-00132). Institutional ethics approvals were also obtained from all participating sites: Ethics Review Committee at Aga Khan University in Karachi, Pakistan (ERC # 1917-OBS-ERC-11); Health Research Ethics Committee at Olabisi Onabanjo University Teaching Hospital in Sagamu, Nigeria (ETHICS # 326/431); Bioethics Committee at Manhiça Health Research Centre in Mozambique (ETHICS # CIBS 05/013); and Institutional Ethics Committee at Karnataka Lingayat Education University’s Jawaharlal Nehru Medical College in India (ETHICS # MDC/IECHSR/2012-13/A-12).

## Results

Refer to CLIP Trial feasibility publications in the current and/or forthcoming supplement.

## Discussion

Feasibility studies are critical to understand the context of intervention prior to clinical trials. Such studies enable researchers to capitalize on facilitators, to remediate barriers, and to tailor operational aspects of interventions in advance of the trial [42]. Moreover, lessons from feasibility studies are instrumental to guide post-trial program scale-up. It is often argued that interventions shown to have promising results in a trial context are not able to be integrated into existing systems post-trial. Therefore, feasibility assessments, guided by robust methods, play a pivotal role in informing the fate of the trial in terms of implementation and post-trial scale-up. According to Lewin, qualitative research is rarely combined with randomized control trials (RCTs), as it was used in only 23 out of 100 RCTs published in English language during 2001–2003 [43]. An exclusively quantitative or qualitative approach cannot appropriately assess the feasibility of a large multi-country community-based clinical trial. A mixed methods design has advantages for validation, contextualization, and triangulation [44].

The mixed method study design used for this study has generated a useful framework which can be employed for future research aiming to evaluate the feasibility of large scale public health interventions. All data collection tools will be made readily available and open access once the primary study results of the trial have been accepted for publication.

**Fig. 8:**
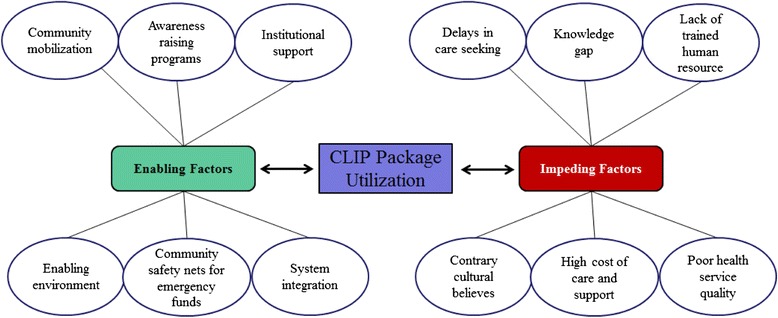
Understanding the context of interventions to maximize the CLIP package utilization. CHWs, community health workers, CLIP, community level interventions for pre-eclampsia; PE/E, pre-eclampsia/eclampsia

The feasibility studies highlight enabling factors including need for community mobilization, awareness raising programs, institutional support, community safety nets for emergency funds, and system integration. Whereas, impeding factors included delays in care seeking, knowledge gaps, lack of trained human resource, cultural myths and misconceptions, high cost of care, and poor health service quality. Lessons learned from this study were used to establish research processes and infrastructure to pave the way for the implementation of the CLIP Trial and post-trial program scale-up should the trial be successful in reducing maternal and perinatal mortality and morbidity (Fig. 8).

Findings also informed local investigators, health practitioners, policy makers, and international research partners on the feasibility of implementing a community level package of care to identify, triage and treat women with pre-eclampsia and eclampsia. Combined FGDs for women of reproductive age- and mothers-in-law, husbands- and fathers-in-law may limit generalizability of study results. First, it could possibly impede open dialogue because of potential cultural barriers whereby young people are unlikely to oppose senior members of the family. Second, it could cause social desirability bias whereby participants respond in a manner that was considered favourable by other family members.

## Conclusions

Feasibility assessments for community level interventions, particularly those involving task-shifting across diverse regions, required an appropriate theoretical framework and careful selection of research methods. The use of qualitative and quantitative methods increased the data richness to better understand the community contexts. The methodological aspects described in this paper can provide guidance for similar studies in other settings.

## Additional file

Additional file 1: Peer review reports. (PDF 305 kb)

## Abbreviations

ANM: Auxiliary Nurse Midwives
APE: Agente Polivalente Elementar
ASHA: Accredited Social Health Activists
CEmOC: comprehensive emergency obstetric care
CHEW: Community Health Extension Workers
CISM: The Manhiça Health Research Centre
CLIP: Community Level Interventions for Pre-eclampsia
CRRH: Centre for Research in Reproductive Health
FGD: focus group discussions
LHW: Lady Health Worker
LMIC: low and middle-income countries
NPT: normalization process theory
PHC: primary health centre
RCT: randomized control trials
SPA: Service Provision Assessment

## References

[1] Steegers EA, von Dadelszen P, Duvekot JJ, Pijnenborg R (2010). Pre-eclampsia. Lancet.

[2] Duley L (2009). The global impact of pre-eclampsia and eclampsia. Semin Perinatol.

[3] Magee LA, Abalos E, von Dadelszen P, Sibai B, Easterling T, Walkinshaw S (2011). How to manage hypertension in pregnancy effectively. Br J Clin Pharmacol.

[4] World Health Organization. WHO recommendations for prevention and treatment of pre-eclampsia and eclampsia. 2011. http://whqlibdoc.who.int/publications/2011/9789241548335_eng.pdf. Accessed 18 March 2015. 23741776

[5] Duley L (1992). Maternal mortality associated with hypertensive disorders of pregnancy in Africa, Asia, Latin America and the Caribbean. BJOG Int J Obstet Gyn.

[6] Hezelgrave NL, Duffy SP, Shennan AH (2012). Preventing the preventable: pre-eclampsia and global maternal mortality. Obstet Gyn Reprod Med.

[7] Bhutta ZA, Darmstadt GL, Hasan BS, Haws RA (2005). Community-based interventions for improving perinatal and neonatal health outcomes in developing countries: a review of the evidence. Pediatrics.

[8] von Dadelszen P, Magee LA, Payne BA, Bhutta Z (2015). The CLIP (Community-Level Interventions for Pre-eclampsia) cluster randomized controlled trial.

[9] Hutcheon JA, Lee T, Magee LA (2011). Using clinical symptoms to predict adverse maternal and perinatal outcomes in women with preeclampsia: data from the PIERS (Pre-eclampsia Integrated Estimate of RiSk) study. J Obstet Gynaecol Can.

[10] Dunsmuir DT, Payne BA, Cloete G, Petersen CL, Gorges M, Lim J (2014). Development of mHealth applications for pre-eclampsia triage. J Biomed Health Inform.

[11] Rouse DJ, Hauth JC, Nelson KG, Goldenberg RL (1996). The feasibility of a randomized clinical perinatal trial: Maternal magnesium sulfate for the prevention of cerebral palsy. Am J Obstet Gynecol.

[12] Korpi-Hyövälti E, Laaksonen D, Schwab U, Vanhapiha T, Vihla K, Heinonen S, Niskanen L (2011). Feasibility of a lifestyle intervention in early pregnancy to prevent deterioration of glucose tolerance. BMC Public Health.

[13] Burrows RF, Burrows EA (1995). The feasibility of a control population for a randomized control trial of seizure prophylaxis in the hypertensive disorders of pregnancy. Am J Obstet Gynecol.

[14] Magee LA, von Dadelszen P, Chan S, Gafni A, Gruslin A, Helewa M, Hannah ME (2007). The control of hypertension in pregnancy study pilot trial. BJOG Int J Obstet Gyn.

[15] May CR, Mair F, Finch T, MacFarlane A, Dowrick C, Treweek S, Montori VM (2009). Development of a theory of implementation and integration: Normalization Process Theory. Implement Sci.

[16] May CR, Mair FS, Dowrick CF, Finch TL (2007). Process evaluation for complex interventions in primary care: understanding trials using the normalization process model. BMC Fam Pract.

[17] May C, Finch T, Mair F, Ballini L, Dowrick C, Eccles M, Heaven B (2007). Understanding the implementation of complex interventions in health care: the normalization process model. BMC Health Serv Res.

[18] Murray E, Treweek S, Pope C, MacFarlane A, Ballini L, Dowrick C, May C (2010). Normalisation process theory: a framework for developing, evaluating and implementing complex interventions. BMC Med.

[19] Denscombe M (2008). Communities of practice: a research paradigm for the mixed methods approach. J Mix Methods Res.

[20] Onwuegbuzie AJ, Leech NL (2005). On becoming a pragmatic researcher: The importance of combining quantitative and qualitative research methodologies. Int J Soc Res Methodol.

[21] Creswell JW. Qualitative inquiry and research design: Choosing among five approaches. Sage. 2012.

[22] Longhofer J, Jacob S (2014). The use of ethnography in social work research. Qual Soc Work.

[23] Guest G, Bunce A, Johnson L (2006). How many interviews are enough? An experiment with data saturation and variability. Field Methods.

[24] Safdar S, Inam SN, Omair A, Ahmed ST (2002). Maternal health care in a rural area of Pakistan. J Pak Med Assoc.

[25] Asowa-Omorodion FI (1997). Women’s perceptions of the complications of pregnancy and childbirth in two Esan Communities, Edo state, Nigeria. Soc Sci Med.

[26] Osubor KOM, Fatusi AO, Chiwuzie JC (2006). Maternal health-seeking behavior and associated factors in a rural Nigerian community. Matern Child Health J.

[27] Furuta M, Salway S. Women’s position within the household as a determinant of maternal health care use in Nepal. Int Fam Plan Perspect. 2006;17–27. 10.1363/3201706 16723298

[28] Bloom SS, Wypij D, Gupta MD (2001). Dimensions of women’s autonomy and the influence on maternal health care utilization in a north Indian city. Demography.

[29] Nordeng H, Havnen GC (2005). Impact of socio‐demographic factors, knowledge and attitude on the use of herbal drugs in pregnancy. Acta Obstet Gynecol Scand.

[30] Borghi J, Sabina N, Blum LS, Hoque ME, Ronsmans C (2006). Household costs of healthcare during pregnancy, delivery, and the postpartum period: a case study from Matlab, Bangladesh. Journal of Health, Population, and. Nutrition.

[31] Britten N (1995). Qualitative interviews in medical research. BMJ.

[32] Lewin S, Munabi-Babigumira S, Glenton C, Daniels K, Bosch-Capblanch X, van Wyk BE, Scheel IB. Lay health workers in primary and community health care for maternal and child health and the management of infectious diseases. Cochrane Database System Rev. 2010;3. 10.1002/14651858.CD004015.pub3 PMC6485809 20238326

[33] Haines A, Sanders D, Lehmann U, Rowe AK, Lawn JE, Jan S, Bhutta ZA (2007). Achieving child survival goals: potential contribution of community health workers. Lancet.

[34] Love MB, Gardner K, Legion V (1997). Community health workers: who they are and what they do. Health Educ Behav.

[35] Rowe AK, de Savigny D, Lanata CF, Victora CG (2005). How can we achieve and maintain high-quality performance of health workers in low-resource settings?. Lancet.

[36] World Health Organization. Maternal, newborn, child and adolescent health. Health facility survey: Tool to evaluate the quality of care delivered to sick children attending outpatient facilities. 2003. http://www.who.int/maternal_child_adolescent/documents/9241545860/en/. Accessed 18 March 2015.

[37] USAID. Health facility assessment. Paiman Pakistan. 2005. http://paiman.jsi.com/Resources/Docs/health-facility-assessment-paiman-original-districts.pdf. Accessed 18 March 2015.

[38] World Health Organization. Service availability and readiness assessment (SARA): an annual monitoring system for service delivery: implementation guide. (2013). Online available http://www.who.int/iris/handle/10665/112798. Accessed 18 March 2015.

[39] USAID. Service provision assessment. 2012. Online available http://www.statistics.gov.rw/survey/service-provision-assessment-spa-survey. Accessed 18 March 2015.

[40] Pedler M. Reflexive methodology: New vistas for qualitative research. Action Learn Res Pract. 2012;83–87.

[41] Wray N, Markovic M, Manderson L (2007). Researcher saturation: the impact of data triangulation and intensive-research practices on the researcher and qualitative research process. Qual Health Res.

[42] De Salis I, Tomlin Z, Toerien M, Donovan J (2008). Qualitative research to improve RCT recruitment: Issues arising in establishing research collaborations. Contemp Clin Trials.

[43] Lewin S, Glenton C, Oxman AD. Use of qualitative methods alongside randomised controlled trials of complex healthcare interventions: methodological study. BMJ. 2009;339. 10.1136/bmj.b3496 PMC2741564 19744976

[44] Spillane JP, Pareja AS, Dorner L, Barnes C, May H, Huff J, Camburn E (2010). Mixing methods in randomized controlled trials (RCTs): validation, contextualization, triangulation, and control. Educ Assess Eval Account.

