# Supplementary material for: The feasibility of community level interventions for pre-eclampsia in South Asia and Sub-Saharan Africa: a mixed-methods design
Source: Reprod Health. 2016 Jun 8;13(Suppl 1):56. doi: 10.1186/s12978-016-0133-0 (PMC4943500; doi:10.1186/s12978-016-0133-0)
Supplement: Additional file 1: — Peer review reports. (PDF 305 kb) [file 12978_2016_133_MOESM1_ESM.pdf]

Referee's comments to the authors– this sheet WILL be seen by the author(s) and published with the article

Title	**The feasibility of community level interventions for pre-eclampsia in South Asia and Sub-Saharan Africa: a mixed-methods design**
Author(s)	Asif Raza Khowaja, Rahat Najam Qureshi, Diane Sawchuck, Olufemi T Oladapo, Olalekan O. Adetoro, Elizabeth A. Orenuga, Mrutyunjaya Bellad, Ashalata Mallapur, Umesh Charantimath, Esperança Sevene, Khátia Munguambe, Helena Edith Boene, Marianne Vidler, Zulfiqar A. Bhutta, Peter von Dadelszen
Referee's name	Isabel De Salis

When assessing the work, please consider the following points, where applicable:

1. Is the question posed by the authors new and well defined?
2. Are the methods appropriate and well described, and are sufficient details provided to replicate the work?
3. Are the data sound and well controlled?
4. Does the manuscript adhere to the relevant standards for reporting and data deposition?
5. Are the discussion and conclusions well balanced and adequately supported by the data?
6. Do the title and abstract accurately convey what has been found?
7. Is the writing acceptable?

Please make your report as constructive and detailed as possible in your comments so that authors have the opportunity to overcome any serious deficiencies that you find and please also divide your comments into the following categories:

- Major Compulsory Revisions (which the author must respond to before a decision on publication can be reached)
- Minor Essential Revisions (such as missing labels on figures, or the wrong use of a term, which the author can be trusted to correct)
- Discretionary Revisions (which are recommendations for improvement but which the author can choose to ignore)

Where possible please supply references to substantiate your comments.

When referring to the manuscript please provide specific page and paragraph citations where appropriate.

General comments:

It makes sense to conduct feasibility studies in diverse locations when trying to run a generic trial and, as the authors state in the abstract, "to understand the facilitators and barriers of new interventions …to tailor operational aspects of the interventions accordingly." A mixed-methods approach seems a good way to undertake such a study. I wish the authors luck conducting the main trial and hope they gleaned useful information in the feasibility study.

However, in this paper as it stands there are no results shown and I am not clear what the aim of this paper is. This is not clearly stated and needs to be. Is it just to report on the methods used in order to help others undertake future community-based trials in this topic area? If this is the case I don't think the methods alone without results can help. As the authors note the methods were labour intensive but without the results showing how useful they were to implement a full-blown trial, and useful to uncover potential problems, there is no way to assess how helpful or not this approach was, its limitations and benefits.

<u>Background</u>

It would be helpful for more general readers to have more details on what the CLIP package is (for example what does the triaging tool consist of) and how it differs from the intervention (how do community healthcare workers provide emergency treatment themselves, and how do they refer to hospital?). I wasn't quite sure whether the intervention was a facilitating or training tool. P. 3 & 4

<u>Methods</u>
The authors seem to have a commendable breadth of methods to assess whether a trial may be doable (part of the feasibility study), and implementable post trial. The methods could do with a clearer structure (Qualitative and Quantitative at least), because at the moment they are confusing, and more succinct. The figures/tables are helpful. The analysis includes points that would be better under the methods section. Few references were given for methodology.

Normalization process theory (not mentioned in abstract). It would be good if the 4 aspects of the framework were explained in more detail and the suggestions included in the study objectives rather than just left as a theoretical framework as if an afterthought. The results could also be looked at in the discussion. The degree the authors found coherence, for example, in the different settings could then be analyzed and discussed. P.6

Some of the expressions are rather puzzling and could do with greater attention to detail: “poor timely access”; is “participatory observation” “participant observation”?; “focus groups discussions” are normally just “focus groups”. (abstract)

Ethnographic approach: this is neither referenced nor well described. We tend to use this term to include qualitative research (e.g. FGs and interviews) that also includes some observational work alongside i.e. participant observation. The authors should either show how their work is ethnographic more clearly and then follow with subsections e.g. focus groups or just keep the section heading as Qualitative followed by subdivisions. P. 7

<u>Focus groups:</u> I liked the mention of how gender was dealt with and I assume “parallel” groups means community members were in different groups to healthcare providers. This makes sense and seems an important point to further briefly expand. P.9
I wasn’t sure what FG “questionnaires” were. Are these like topic guides? P11.
There seem to be some blurring when the authors describe “cognitive participation”. Were focus groups and interviews primarily about trying to elicit existing understandings and practices (as in the objectives) including those which may promote or hinder the implementation of the intervention, or were they also about informing people of the intervention/trial or trying to promote the intervention? P.6

<u>Discussion</u>
A “feasibility study” is done to assess whether a full blown trial can be done. Of course, when collecting data it is worthwhile to look at the related but different point about post trial generalizability, but this needs to be considered a separate point and not merged together.

The results in figure 8 need to much further developed showing the data to support these findings, whether they were all the same across the diverse locations and how it impacted or will impact in the running of the trial. Reference to the 4 aspects of normalization process theory could also be referred to, and indeed could provide a framework for the results.

It would also be interesting to note what differences the authors found in the different contexts and how this impacted on how the trial was to be run in diverse cultural locations. Many people are trying to run generic trials often with little reference to local context. The authors could make this much more evident.

This paper needs major revisions to be publishable.

Referee's comments to the authors– this sheet WILL be seen by the author(s) and published with the article

Title	**The feasibility of community level interventions for pre-eclampsia in South Asia and Sub-Saharan Africa: a mixed-methods design**
Author(s)	Asif Raza Khowaja, Rahat Najam Qureshi, Diane Sawchuck, Olufemi T Oladapo, Olalekan O. Adetoro, Elizabeth A. Orenuga, Mrutyunjaya Bellad, Ashalata Mallapur, Umesh Charantimath, Esperança Sevene, Khátia Munguambe, Helena Edith Boene, Marianne Vidler, Zulfiqar A. Bhutta, Peter von Dadelszen
Referee's name	Katie Birdsall

When assessing the work, please consider the following points, where applicable:

1. Is the question posed by the authors new and well defined?
2. Are the methods appropriate and well described, and are sufficient details provided to replicate the work?
3. Are the data sound and well controlled?
4. Does the manuscript adhere to the relevant standards for reporting and data deposition?
5. Are the discussion and conclusions well balanced and adequately supported by the data?
6. Do the title and abstract accurately convey what has been found?
7. Is the writing acceptable?

Please make your report as constructive and detailed as possible in your comments so that authors have the opportunity to overcome any serious deficiencies that you find and please also divide your comments into the following categories:

- Major Compulsory Revisions (which the author must respond to before a decision on publication can be reached)
- Minor Essential Revisions (such as missing labels on figures, or the wrong use of a term, which the author can be trusted to correct)
- Discretionary Revisions (which are recommendations for improvement but which the author can choose to ignore)

Where possible please supply references to substantiate your comments.

When referring to the manuscript please provide specific page and paragraph citations where appropriate.

General comments:

- **Some minor essential revisions. A few sections of the paper could have the written tightened up and grammar either corrected or made more consistent throughout the paper. Tables and figures are fine, but double check Figure 2 (see below).**

Major compulsory revisions:

- **None noted**

Minor essential revisions:

- **Figure 2 – "Cross-cutting Priory Themes" – should it not be "Cross-cutting Priority Themes"?**
- **Clarification needed**
    - **Line 263. Researcher instead of research?**
- **Line 280-1. Have already introduced CEmOC as an acronym. Use instead.**
- **Line 386-388. Data from 2003. Still the trend?**

(continue on the next sheet)

Continued:

Discretionary revisions:

- **Abstract**
    - **Lines 10-11. State ability to potentially scale up as a benefit**
    - **Lines 72-4. Discusses the rarity of feasibility assessments. This seems important. Bring into abstract**
- **Clarification needed**
    - **Line 184-5. Community representatives versus participants?**
- **Acknowledgement in either Data Collection section or the Discussion section about potential biases.**
    - **Lines 185-6. Discusses the participation of decision-makers who accompanied women – perhaps cannot generalize to all decision-makers?**
    - **Lines 200-201. Any bias in choose pregnant women and women with children under 5 as representative of women of reproductive age? (In countries selected, perhaps not…)**
- **Grammar / punctuation:**
    - **Lines 66-7. Clarify "… delivery systems, and a diversity…"**
    - **Line 92. "… to assess each health care…"**
    - **Lines 125-132. " … one another and allow for…"; "and practices, as well as…"; "and skills, as well as health…"**
    - **Line 137. "… constructed, influences…"**
    - **Line 143. "… complications with…"**
    - **Line 153. "… country, as well as existing…"**
    - **Line 167. "One researcher, assisted by a second to record field notes and audio recordings, facilitated…"**
    - **Line 182. "… for the study if they…"**
    - **Line 188. "… the community in a way…"**
    - **Line 191. "… to participate and arranged…"**
    - **Line 195. "… refreshments and provided…"**
    - **Line 204-207. "… particularly mothers…"; "… countries: Community…"; "… (ASHA), staff nurses…"**
    - **Line 225. "… appropriate. This…"**
    - **Line 236. "… interviews if they…"**
    - **Line 268. "Systematic reviews of…"**
    - **Line 276-285. "… resources and capabilities…"; "… basic, as well as comprehensive…"; "… research team and questions…"**
    - **Line 297-308. "… therefore…"; "… content prior to…"; "… informed consent…"; "… Likert scale. This…"; "… or home to minimize…"; "… the questionnaire unless…"**
    - **Line 323. "… included field…"**
    - **Line 330. "… data, as well as ensuring…"**
    - **Line 343-351. "… FDGs were…"; "… multiple researchers with…"; "Each FDG and interview…"; "…deductive approaches)…"**
    - **Line 371. "… Sagamu, Nigeria…"**
    - **Line 396. "… processes and infrastructure…"**

Supplement Editor comments:

Please mainly consider one of the reviewers´ concern about the deficiency on providing results on this manuscript. The manuscript should be read as an independent article without the need to go over the other articles of the Supplement.
